# Discordance between invasive coronary flow reserve (CFR) and non-invasive myocardial flow reserve (MFR) in diffuse coronary ectasia (DCE)

**DOI:** 10.1259/bjrcr.20190046

**Published:** 2020-09-29

**Authors:** Mohammed Khalil, Deniz Alibazoglu, Cem Numan Balci, Rawan Hussein, Abraham Abuwadi, Haluk Alibazoglu

**Affiliations:** 1 Heart and Vascular Institute, Cleveland Clinic Abu Dhabi, Abu Dhabi, UAE; 2 Imaging Institute, Cleveland Clinic Abu Dhabi, Abu Dhabi, UAE

## Abstract

Clinical use with evidence base for diagnostic and prognostic value of quantitative positron emission tomography(PET) myocardial perfusion imaging (MPI) in patients with known or suspected coronary artery disease has exponentially increased over the last decade. This case illustrates the very first time that stress myocardial blood flow(MBF) in absolute terms (ml/min/gram) and myocardial flow reserve(MFR) are augmented in three vessel diffuse coronary ectasia by N13-Ammonia PET MPI. Moreover, relative qualitative MPI demonstrated moderate-sized ischemia in right coronary artery territory with chronic total occlusion in middle segment; despite regional myocardial flow reserve remains above ischemic thresholds while regional stress myocardial blood flow is mildly reduced.

## Case presentation

**Figure 1.**
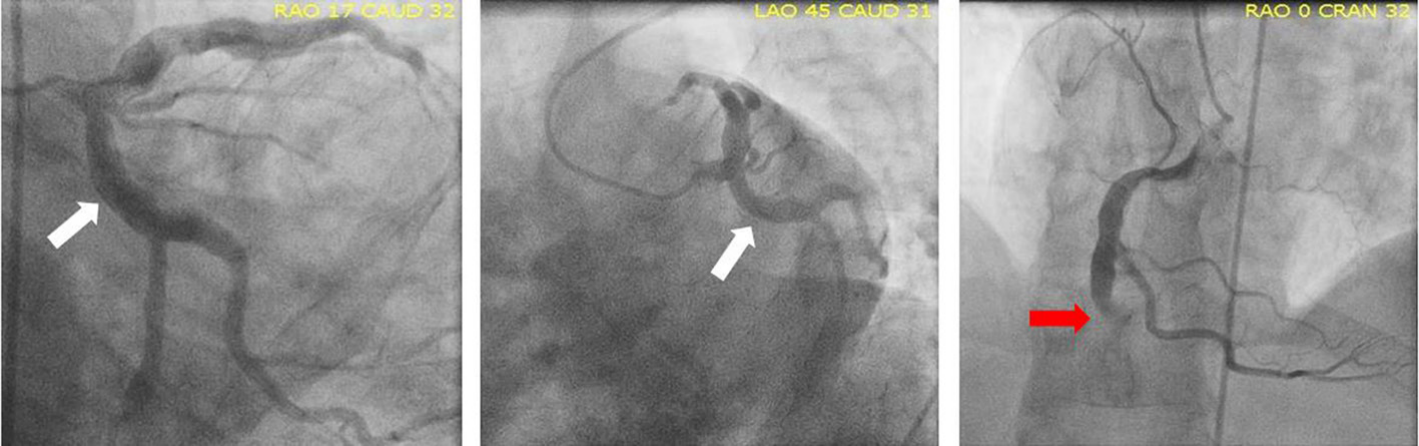
Three vessel DCE Type I with LCX (white arrow) was the most severe. Mid-RCA had CTO (red arrow). CTO, chronic total occlusion; DCE, diffuse coronaryectasia; LCX, left circumflex artery; RCA, right coronary artery.

Asymptomatic, 43-year-old, hypertensive male with a body mass index of 45 was referred to positron emission tomography myocardial perfusion imaging (PET MPI) for risk stratification. He had a history of inferior myocardial infarction (MI) 1.5 years ago followed by unsuccessful percutaneous coronary intervention to chronic total occlusion (CTO) of middle right coronary artery (RCA) with high thrombus burden. He was found to have significant three vessel diffuse coronary ectasia (DCE) with the most prominent in the left circumflex artery (LCX) (Figure 1).

## Investigations

Following strict abstinence from caffeine-containing products for 24 h and establishment of i.v. access in a basilic vein in the antecubital fossa, dynamic rest myocardial perfusion PET/CT imaging was performed by injection of 15.8 mCi of NH13-Ammonia. Dynamic stress myocardial perfusion PET/CT imaging was performed 120 min later, with injection of 0.4 mg Regadenoson followed 55 seconds later by injection of 15.9 mCi NH13-Ammonia. 75 mg of i.v. aminophylline was administered at 120 seconds after Regadenoson flush was completed. All acquisitions were performed by Siemens Biograph mCT (64) 3R (Siemens Medical Solutions USA Inc., Hoffman Estates, IL). For accurate sampling and integration of the arterial blood activity, sequential short time frames were applied during dynamic imaging (24 frames/5 s, 6 frames/10 s, 6 frames/30 s and 5 frames/60 s). Rate pressure product (RPP) data were acquired at every minute in the first 5 min of rest and stress acquisitions and averages were calculated. During the vasodilator stress procedure, no symptomatic, hemodynamic or ECG evidence of ischemia were noted. Rest ECG was consistent with inferior MI.

All three pairs of image data sets (static, dynamic and gated) for rest and stress were processed and analyzed by using an automated program (Corridor4DM; Invia, Ann Arbor, MI) utilizing 1-tissue compartment model (INVIA N-13 ROI 1:1) and displayed with the standardized 17 segment model. Manual inspection and correction of misregistration of CT and PET data sets were also performed prior to processing. For quantification of rest and stress MBF and calculation of MFR, motion correction of individual frames in the dynamic data sets were manually performed to minimize spill over into tissue and blood pool compartments.

**Figure 2.**
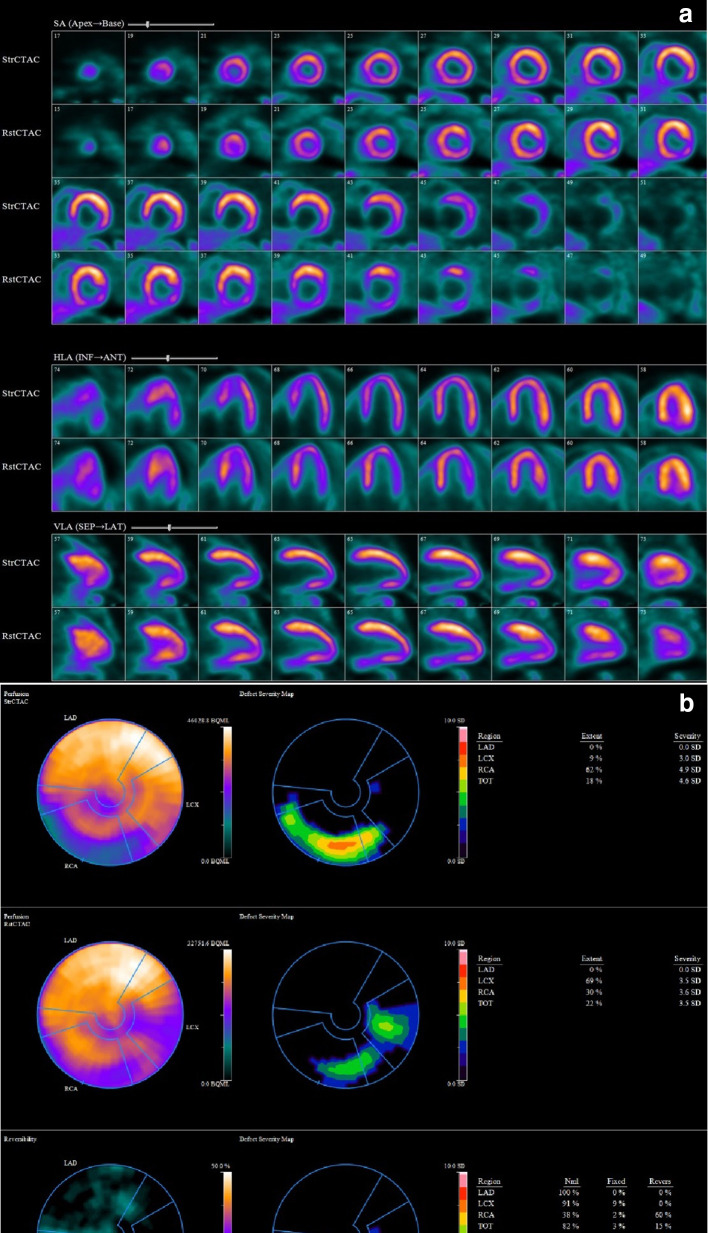
(a, b) Rest and stress regadenoson PET MPI demonstrated ischemia of moderate size (15% of LV myocardium) in inferior wall and a very small fixed perfusion abnormality (3% of LV myocardium). LV, left ventricle; MPI, myocardial perfusion imaging; PET, positron emission tomography.

Qualitative relative PET images demonstrated a partially reversible defect in inferior wall of moderate size (18% LV myocardium) and moderate severity, with small peri-infarct ischemia (less than 10% of LV myocardium) consistent with non-transmural MI (Figure 2A,B). Estimated left ventricular ejection fraction was 62% in stress and 65% in rest; consistent with lack of augmentation of systolic function and impaired left ventricular ejection fraction reserve. Overall left ventricular systolic function was normal without any wall motion abnormalities.

**Figure 3.**
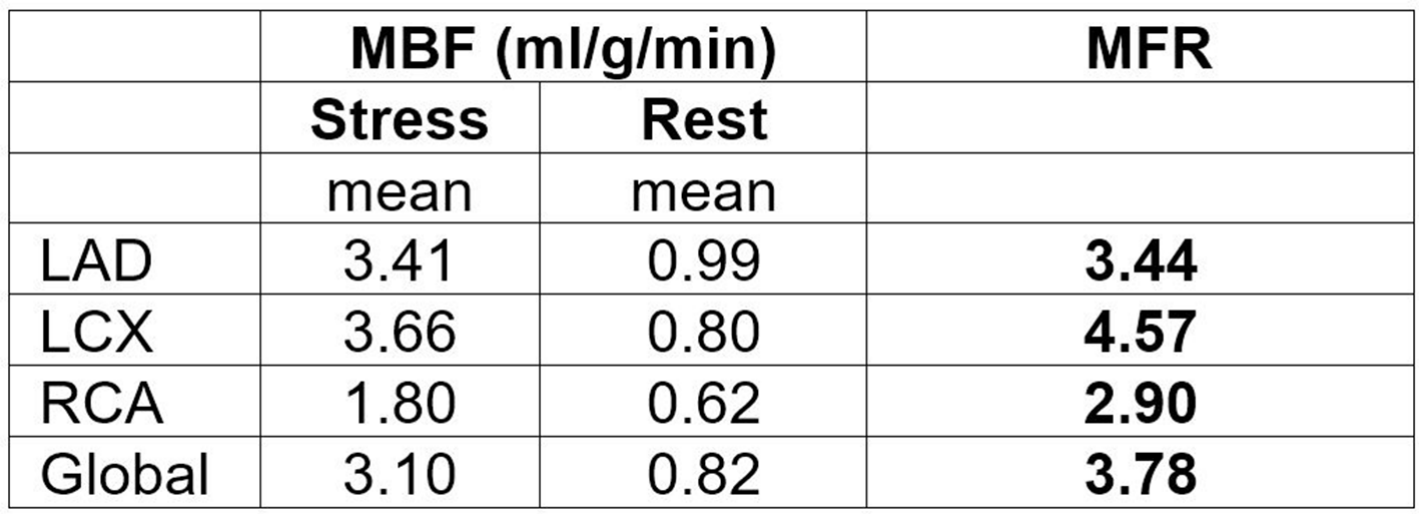
MFR was preserved (above 2.0) globally and in individual three coronary territories; LCX with the most severe ectasia demonstrated the highest MFR and stress MBF. Totally occluded RCA with ischemia in qualitative relative MPI had relatively lower stress MBF (1.80 ml/min/g) compared to other territories but preserved MFR (2.90). LCX, left circumflex artery; MFR, myocardial flow reserve;MPI, myocardial perfusion imaging; RCA, rightcoronary artery.

Calculated average RPP was 10,394 in rest and 12,998 in stress. Rest MBF was calculated without application of RPP adjustment (10.000) to the rest MBF data. All three coronary territories demonstrated preserved to high MFR; LCX being the most severely ectatic had the highest MBF and MFR values followed by LAD. Totally occluded RCA had relatively the lowest values with mildly reduced stress MBF but preserved MFR (Figure 3).

## Follow-up

The patient has been asymptomatic since 16 months under maximum guideline-directed medical therapy and repeat PCI for CTO of RCA was not attempted.

## Discussion

Coronary artery ectasia is defined as dilation ≥1.5 fold the normal vessel diameter^1^ . A proposed theory for the mechanism of luminal dilation is arterial remodeling with expansion of the tunica media and external elastic membrane due to chronic severe inflammation^2^ . Association of angina, positive exercise stress test and acute coronary syndromes have all been attributed to decreased coronary flow velocity in DCE^3^ . Hence, coronary flow velocity measurements in epicardial arteries by invasive Doppler method, with the ratio of stress/rest flow velocity as coronary flow reserve (CFR) was shown to be significantly reduced in patients with DCE^4^ . For flow velocity measurements at peak stress, papaverine was used as a coronary vasodilator; acting on smooth muscle cells located in tunica media.

In comparison to epicardial flow velocity measurements by invasive Doppler method, quantitative PET MPI non-invasively measures MBF within the myocardial tissue in absolute terms (ml/min/g), reflecting an integrated composite value of MBF in the entire coronary vascular tree; epicardial and microvascular beds with the ratio of stress/ rest MBF as MFR. Peak stress is achieved by utilizing the most commonly used vasodilator agent regadenoson; selective agonist of A2A receptors located in the vascular endothelium in tunica intima.^5^

It has been shown that non-invasive assessment of MFR by NH13-Ammonia and Rubidium-82 imaging generally correlates well with invasively determined CFR.^6^ However, our case illustrates that in patients with DCE, this relation doesn’t appear to hold true. In contrast to previously demonstrated attenuated stress flow velocity with significantly reduced CFR in patients with DCE,^4^ our case demonstrated augmented global stress MBF with markedly increased global MFR with regadenoson. To our knowledge, in patients with DCE, this is the first case in the literature demonstrating augmented vasodilator flow response with markedly elevated global stress MBF and MFR by quantitative PET MPI. The perfusion bed of LCX; the most ectatic coronary vessel had the highest stress MBF with the highest MFR. It was also noteworthy that in the relative perfusion images in rest, there was an artefactual perfusion defect in LCX territory which was not apparent at stress; most likely associated with normal variant lateral wall perfusion abnormality previously demonstrated in NH13-Ammonia studies^7^ .

Given excessive expansive vascular remodeling with enzymatic degradation of the extracellular matrix of the tunica media to be a fundamental pathologic process in coronary ectasia,^8^ as our case demonstrates, tunica intima mediated vasodilator capacity remains intact which operates via A2A receptor-mediated nitric oxide production in intimal endothelial cells.^9^ Moreover, not only preserved but also augmented vasodilator capacity with very high MFR values were noted in our case. A potential mechanism can be conceptualized by an increase in the vessel luminal surface area in DCE with more availability of tunica intima where regadenoson operates on A2A receptors resulting in higher nitric oxide release. It is also noteworthy that given diffuse dilation ≥1.5 fold the normal vessel diameter, global rest MBF remains within normal physiologic range^6^ and doesn’t appear to confound the MFR calculation.

As our case illustrates, in the setting of DCE, flow velocity based measures of CFR and absolute flow based measures of MBF are discordant, not interchangeable and has the potential for confusion; given the fundamental differences between what is being measured by each technique, as well as strengths and limitations of these physiologic parameters for clinical decision making. Moreover, the term MFR has also been considered more appropriate for PET measures of myocardial flow in recent multisociety joint position paper in clinical quantification of MBF using PET.^6^

It has been well documented that severe reduction in stress MBF (<1.5 ml/min/g) and /or MFR (<1.5) in a single vascular territory may indicate regional flow-limiting coronary artery disease.^6,10^ Contrarily in our case, the totally occluded RCA territory had preserved MFR of 2.9 and mildly reduced stress MBF (1.80 ml/min/g) despite with moderate-sized ischemia in relative qualitative MPI. It was noteworthy that preserved MFR was not a result of very low rest MBF due to scarred myocardium which typically demonstrates rest MBF of 0.2 ml/min/g.^11^ It was most likely that the downstream totally occluded territory had well established collateral flow; maintaining normal rest MBF of 0.62 ml/min/g (range 0.4–1.2 ml/min/g)^6^ and hence viability of the jeopardized territory. However, the well established collateral flow was not sufficient enough to achieve a preserved stress MBF above 2.0 ml/min/g in the setting of CTO; resulting in mildly reduced stress MBF (1.80 ml/min/g) and moderate-sized ischemia in MPI.

The impact of CTO in RCA territory was a perfusion heterogeneity with resultant ischemia in MPI. Regional heterogeneity of quantitative flow measures were also discernable when RCA territory was compared with the other patent two territories. RCA territory reveals relatively much lower stress MBF and MFR. Given the patient being asymptomatic and with no major adverse events during a follow-up of 16 months, preserved to increased global and regional MFR is likely associated with good prognosis in the setting of DCE.

## Learning points

1. The case illustrates the very first time that PET measures of global and regional MFR are significantly augmented in DCE due to markedly increased coronary vasodilator capacity and stress MBF.
2. Although flow velocity based invasive CFR and absolute flow based non-invasive MFR generally correlate well, these measures of coronary physiology are discordant in DCE due to the fundamental difference of the techniques, flow velocity versus absolute flow measurements. In contrast to a previous report of significantly reduced flow velocity and CFR, this case illustrates markedly elevated stress MBF and MFR in a patient with DCE.
3. Given the primary pathophysiological process involves tunica media in DCE, this case illustrates that tunica intima mediated vasodilator capacity is intact and responsible for the augmented response to regadenoson and global MFR.
4. In the setting of DCE, a territory with CTO demonstrated preserved regional MFR likely due to well-established collateral flow with intact vasodilator capacity albeit evidence of perfusion and absolute flow heterogeneity.
